# From What Age Is Mental Rotation Training Effective? Differences in Preschool Age but Not in Sex

**DOI:** 10.3389/fpsyg.2018.00753

**Published:** 2018-05-16

**Authors:** Laura M. Fernández-Méndez, María José Contreras, M. Rosa Elosúa

**Affiliations:** Departamento de Psicología Básica I, Universidad Nacional de Educación a Distancia, Madrid, Spain

**Keywords:** spatial skills, mental rotation, spatial training, sex differences, spatial development, preschool education

## Abstract

Currently, there is evidence that spatial skills training leads to an improvement of such skills, although studies with children in the Preschool stage are very scarce. This paper aims to examine the effectiveness of mental rotation (MR) training and sex differences in preschool children. Two experiments were carried out. Experiment 1 included 59 children of 1st course (aged between 3 and 4 years) and Experiment 2, 61 children of 2nd course (aged between 4 and 5 years) of Preschool Education, distributed into control and training groups. The results showed a significant improvement in the MR ability of the training group (measured through a different test than the one used for training) only in the older children, and a tendency toward significance in the younger participants. Moreover, no sex differences in MR or group differences across age groups were found. These results regarding MR training support the malleability of spatial skills approach, particularly in 4–5 year-old preschoolers. This malleability should be enhanced in our educational system, as well as the implementation of educational and social policies that tend toward equality between sexes in the development of spatial skills. This can promote an equitable access to academic careers requiring high spatial skills such as engineering, science, technology or mathematics, in which nowadays women are underrepresented.

## Introduction

Mental rotation (MR), one of the most studied spatial skills within spatial cognition, has been defined as a dynamic process which requires mentally rotating a stimulus in order to align it with another reference stimulus, judging whether both stimuli are the same (Shepard and Metzler, 1971). A considerable increase in the research of this ability has occurred since the early 70’s (Shepard and Metzler, 1971; Cooper and Shepard, 1973); however, studies focused on children younger than 6 years of age are very scarce, leading to the emergence of a field of research centered on when and how this ability develops. In this regard, several studies show the existence of MR processes in 5 year old children (Funk et al., 2005; Frick et al., 2009) and even in children as young as 4 years and 6 months of age (Marmor, 1975), whereas other studies have found no signs of MR in children under 5 years old or have only found a small proportion of children that seem to apply MR strategies (Estes, 1998; Noda, 2010; Frick et al., 2013a,b; Hawes et al., 2015). Regarding research on 3 year old children, some authors, such as Krüger et al. (2014), have found mental transformation processes analogous to those found in older children or adults in a task that required the use of MR. However, other authors such as Frick et al. (2013b) observed perseverative patterns in their responses without signs of MR, where less than half of the 4-year-old children exceeded chance performance level. These findings show how MR skills change at such an early age, resulting in inconsistent results and individual differences being reported.

Given the importance of spatial skills in many activities of daily life (Uttal et al., 2013b) such as environment learning (Pazzaglia and Meneghetti, 2012), academic competences like science and mathematics (Laski et al., 2013) or motor skills (Moreau et al., 2012; Oudgenoeg-Paz et al., 2014), it seems particularly important to establish whether such skills are modifiable and at what age do they develop. Uttal et al.’s (2013a) meta-analysis has shown the high malleability of spatial skills, whose training results in an effective, durable and transferable improvement. It is noteworthy that only 53 out of 206 studies were performed on children under the age of 13 years. In relation to primary school children, Tzuriel and Egozi (2010) carried out a training to improve representation and transformation of visuospatial information in 6 year old children. In this age group, the trained group showed an improvement in their MR ability with respect to the control group, yielding evidence of MR malleability through training of spatial ability. In the Preschool stage, several studies have shown that early experiences with games with a visuospatial content, such as building blocks in children between 4 and 6 years of age (Casey et al., 2008) or puzzle making between the ages of 2 and 4 years, can alter spatial skills (Levine et al., 2012). As for MR training, in Frick et al.’s (2013a) study, children aged between 4 and 5 years were trained in a task where they had to decide whether a piece (rotated or mirrored), situated on the top of a board, fitted into a hole at the bottom of the board. A total of 48 children, who were administered 18 training trials, were assessed. The results showed a training effect only in 5 year-olds, with no improvements being found in 4 year-old children, who did not benefit from training. Similar results were obtained by Ehrlich et al. (2006), whose data showed improvement in 5 year-old children who received 12 training trials (of which only 6 were of MR). In this task, the participant observed the two parts of a shape in order to later point out in a matrix containing four different figures, which one represented the union of the two parts.

Moreover, some studies have failed to find effects of MR training in preschool children, such as Marmor’s (1977) work with 4 and 5 year old children. The participants of the training group in that study received seven rotation training trials in which they had to decide whether the two figures, one being either a rotated mirror image or a rotation across the plane of the other, were the same or different from each other. However, the control group performed a similar but slightly different task than the training group, where they were shown seven pairs of stimuli, the images appeared unrotated and the experimenter rotated them in front of the child, who then decided whether both images were the same or not. Both groups performed similar rotation tasks in the training stage; the difference lay in the presentation of stimuli and on whether the rotation was performed in front of the participant or not. The fact of not finding a training effect for the training group, compared to the control group, could be because the latter also performed a similar task with stimuli in rotation that could be considered a type of training. These results raise the debate on the age at which MR training can achieve effective results; the fact that only three studies (Marmor, 1977; Ehrlich et al., 2006; Frick et al., 2013a) have explored the effect of MR training on preschoolers without finding an obvious result as to the age at which MR can be trainable, linked with studies showing that children have the capability to make mental transformations at the age of 3 years (e.g., Krüger et al., 2014), suggest the need to study the effect that a more specific and adapted training could have at these early stages of development. Therefore, more research is needed to further delve into this issue. For this reason, the present work focused on the age at which MR training can be effective.

Another objective of this present study was to analyze possible differences between sex^1^ groups at such early stages of development. Numerous previous studies have consistently shown sex differences in MR, with a greater group average performance for men compared to women within the adult population (Reilly and Neumann, 2013; Debelak et al., 2014; Voyer and Jansen, 2016). In this sense, Linn and Petersen (1985), in their meta-analysis, established significant mean differences between sex groups in MR only in adults, as no studies with children under the age of 10 years were included. However, in a subsequent meta-analysis (Voyer et al., 1995) that included MR studies on children under the age of 10 years, no significant differences between sex groups were found in three of the four studies (Caldwell and Hall, 1970; Kaess, 1971; Jahoda, 1979). The results of Voyer et al.’s (1995) meta-analysis and other previous works (Caldwell and Hall, 1970; Kaess, 1971; Jahoda, 1979) have shown a positive relationship between chronological age and effect size, suggesting that the mean differences between sexes increase with age. Thus, a crucial question arises as to the time of development in which these differences emerge. Several studies have sought to investigate this issue, although there are conflicting results with evidence in both directions for the Preschool stage. Levine et al. (1999) found mean differences between sex groups only for children aged 4 years and 6 months onward, in a mental transformation task which required some MR operations. On the contrary, the studies that have not found these differences in Early Childhood Education (Platt and Cohen, 1981; Estes, 1998; Frick et al., 2009) are more numerous. It seems that the average differences between sex groups, in terms of MR, start after the Preschool stage (Johnson and Meade, 1987; Titze et al., 2010), which could indicate the decisive influence of the environment and the social context, as well as the possibility of changing this path in a direction that is not discriminative toward females.

In the present study, our first objective was to evaluate the effectiveness of a MR training program in the first 2 years of the Preschool stage. The first hypothesis posed that the training group would achieve a significantly higher mean increase than that of the control group in a different MR task to that used in training. Other studies have found an increase of performance in MR skills after training in adults (Wright et al., 2008; Meneghetti et al., 2016a), however, to our knowledge, there are only three studies with preschoolers and their results are inconsistent (Marmor, 1977; Ehrlich et al., 2006; Frick et al., 2013a). Moreover, at the age of 3 years, it seems that children can successfully perform tasks of spatial transformation (Krüger et al., 2014). Our second objective analyzed the possible average differences between sex groups in MR. As there are studies that have yielded evidence in both directions, those that have found sex differences (Levine et al., 1999) and those that haven’t found any differences between males and females at this early stage (Frick et al., 2009), a clear hypothesis cannot be formulated regarding sex differences because previous finding are inconsistent. Moreover, as there are two meta-analyses that have indicated that males and females benefit equivalently from MR training (Marulis et al., 2007; Hand et al., 2008), improvements for both sexes in the MR test may be observed.

To fulfill these objectives, we carried out two experiments. In Experiment 1, we tested both objectives on children enrolled in first course of Preschool Education (3 and 4 year-olds) and in Experiment 2, the same objectives were tested on preschoolers enrolled in second course (4 and 5 year-olds).

## Experiment 1

### Materials and Methods

#### Participants

In this study, 59 students of 1st year of Preschool Education from a school with a medium-high socioeconomic level took part. The students were randomly distributed into two groups (training group and control group). The tests were always applied by the same experimenter. Throughout the course of the study, one participant was excluded for not performing some of the tests, finally leaving the training group with 29 participants (15 boys and 14 girls; *M* = 3.68 years and *SD* = 0.28) and the control group with 29 participants (16 boys and 13 girls; *M* = 3.58 years and *SD* = 0.32). This experiment was carried out in accordance with the recommendations of The Ethics committee of the University (UNED). The protocol was approved by the Ethics committee of the University (UNED). Parents were contacted through their children’s schools, and written consent was obtained from them, in accordance with the Declaration of Helsinki.

#### Materials

##### Abbreviated Picture Rotation Test

This test was adapted from the *Picture Rotation Test* (Quaiser-Pohl, 2003; Marke, 2008) to fit the level of 3–4 year old children. The original task consisted of 16 sheets containing color images of animals and people, with three possible answers. As the original version of the PRT was oriented to the evaluation of MR in 4–6 year-old children, it had to be modified in order to adapt its difficulty to the level of 3 year-old children. These modifications were suggested by its author, Dr. Quaiser-Pohl through a personal communication. This was achieved by reducing the number of trials, the response options and the angles of rotation to apply it to children from 3 years of age onward. Thus, the adapted version of the PRT had five practice trials and eight evaluation trials with two response options. The adaptation was tested through two pilot studies in which the adequacy of this test was evaluated for 3 and 4 year olds. This guaranteed that this adaptation was a valid measure of MR ability. In the practice trials, the figures that were to be mentally rotated could also be manipulated by the experimenter (in the case of the first trial) or directly by participants (in the remaining trials), while, in the evaluation trials, the figures from the sheets could not be manipulated. The rotation angles of the figures were 45° in half of the trials and 180° in the other half of trials.

The design of the sheet presented a non-rotated figure on the left side, separated with a black line from another two figures located in the right area, which were rotated in the plane or a rotated mirror image of the figure situated on the left hand side. The participant’s task was to choose which of the two figures matched the figure on the left. The approximate duration of the task was 10–15 min. One point was awarded for each correct choice, with the maximum possible score being 8 points. This test obtained a test – retest reliability index of 0.52 and 0.59 in the training and control groups, respectively.

##### Mental Rotation Training

To carry out the training, material used in several previous studies related to MR in preschoolers (Frick et al., 2013b) was chosen, although it was slightly modified. The same types of figures (ghosts) made by Frick et al. (2013b) were used. However, in this study, specific rotation settings for each of the training sessions, different from those presented by the original authors, were used.

The training consisted of three sessions of increasing difficulty both, across and within sessions, applied on consecutive days. The increase in difficulty was determined by the angular disparity, so that greater difficulty was associated with an increased rotation angle of the stimuli presented. The training consisted of 62 sheets. At the beginning of each session, one practice trial without rotation (only translational move) and four practice trials (with the same angular disparity than those later trained) were presented in order to familiarize the child with the task. As the first session trained 0° the practice only had four trials. In the first session, angles of 0°, 30°, 60°, and 90° were trained (with 4 practice trials and 16 training trials); in the second session, angles of 30°, 60°, 90°, and 120° were trained (with 5 practice trials and 16 training trials); in the last session, angles of 90°, 120°, 150°, and 180° were trained (with 5 practice trials and 16 training trials). Each angular disparity was trained four times in each session.

**FIGURE 1:**
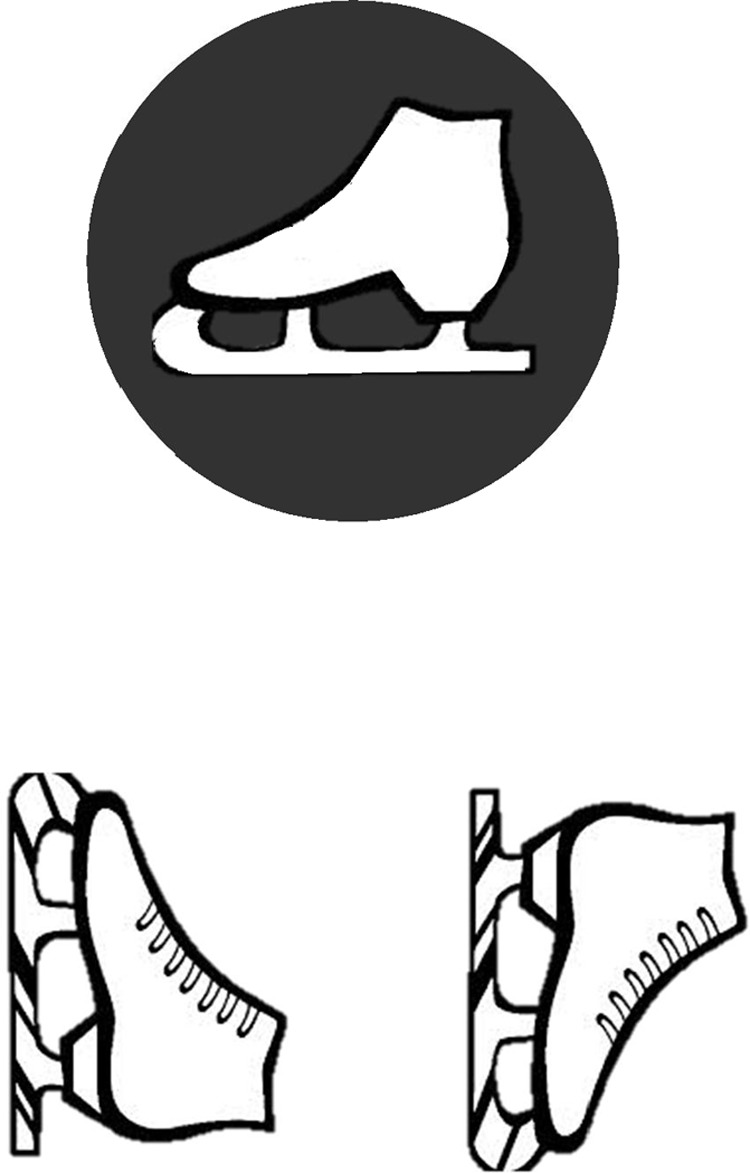
Example of a practice trial.

Each sheet (size 215 × 315 mm) contained three figures, two of them were located at the bottom of the sheet and were named as “1” and “2,” depending on their arrangement with respect to the margin, being “1” the closest to the left margin of the page and “2” the closest to the right margin. The third figure, located on top of the sheet, was a black circle containing a white figure as template (outline only) with the same shape as figures “1” and “2,” and was termed as reference figure. Figures “1” and “2” had the same degree of rotation, one being the rotated image (R) and other being the mirror image (E) of the reference figure. The figures in the practice sheets were common objects (for example, see **Figure 1**), while the figures of the training sheets were ghosts of different shapes, created by Frick et al. (2013b).

#### Procedure

The application of the test was distributed in three phases: pretest phase, training and post-test phase. All participants performed the *Abbreviated Picture Rotation Test* on the pretest and post-test phase. In the training phase, while the training group was trained, the control group attended their classes as usual. The time elapsed between pretest and post-test was approximately 2 months for both groups. The break between pretest and post-test was due to the high time cost of the pretest phase, as the assessment was carry out individually, one child at a time, with brief sessions carried out exclusively by a single experimenter. Moreover, testing had to be carried out adjusting to school timetables, with no assessments being carried out during the hours in which the children had free time/recess, any special activities, gymnastics/P.E. or English classes. The trained group started the post-test the next day or 2 days after finishing the training. The test was performed individually (due to the age of the participants) to avoid distractions and to increase concentration, in a classroom enabled for this purpose. Each child took approximately two sessions of 15 min to complete the pretest and the post-test.

The training phase consisted of three sessions administered on consecutive days, approximately 15 min in length each, with a total duration of 45 min of training. The application was performed individually and manipulatively, where once the child had chosen the figure he/she considered to be correct, he/she was asked to rotate and fit it into the mold (reference figure), hence receiving feedback as to the correctness of his/her responses, which was confirmed orally by the experimenter. If the figure fit the reference mold, the next trial would commence; if the incorrect figure was chosen and it did not fit into the mold, the child was asked to manipulate the other figure into place. The participants were asked to choose correctly before handling the selected figure and their answer was recorded once the figure had been handled for all trials, including practice and training trials, as it was noted that some participants adverted they were making a mistake when slightly moving one figure and would quickly change to the correct figure.

### Results

The results of the MR scores in the control and training groups are shown in **Table 1**.

**Table 1:** Mean (standard deviation) and effect size (Cohen’s *d*) of mental rotation measures in the pretest and post-test and the pre-post-test differences (increases) for control group and training group in 1st course of Preschool Education.

	Pretest	Post-test	Increase	*d*
Control group (*N* = 29)	4.75	5.32	0.57^∗^	
	(1.17)	(1.25)	(1.1)	
				*0.50
Training Group (*N* = 29)	4.96	6.11	1.15^∗^	
	(1.02)	(1.4)	(1.23)	

*^∗^*p* < 0.05, ^∗∗^*p* < 0.01.*

In order to contrast hypotheses 1 and 2, a mixed-model repeated measures ANOVA was applied: 2 group (training vs. control) × 2 sex (males vs. females) × 2 time (pretest vs. post-test), with the first two factors as between-subjects and the last factor as within-subjects. The results revealed a significant main effect due to time, *F*(1,51) = 28.631, *p* = 0.000, $\eta_{p}^{2}$ = 0.36, and a non-significant interaction between time and group, *F*(1,51) = 3.53, *p* = 0.073, $\eta_{p}^{2}$ = 0.062, although it showed a bias toward significance. This analysis showed a significant interaction between group and sex, *F*(1,51) = 4.99, *p* = 0.030, $\eta_{p}^{2}$ = 0.089 for the between-subjects effects. It is noteworthy that this effect was not present in intrasubjects measures. Thus, it can be concluded that sex did not influence the training. However, to clarify this interaction, it was confirmed that it appeared because had girls outperformed boys in the control group and boys had outperformed girls in the training group in the pretest (within the CG, males with *M* = 4.47, *SD* = 0.99, and females with *M* = 5.08, *SD* = 1.32; within the TG, males with *M* = 5.31, *SD* = 1.18, and females with *M* = 4.64, *SD* = 0.75) and in the post-test (within the CG, males with *M* = 5.13, *SD* = 1.19, and females with *M* = 5.54, *SD* = 1.33; within the TG, males with *M* = 6.54, *SD* = 1.33, and females with *M* = 5.71, *SD* = 1.38).

**FIGURE 2:**
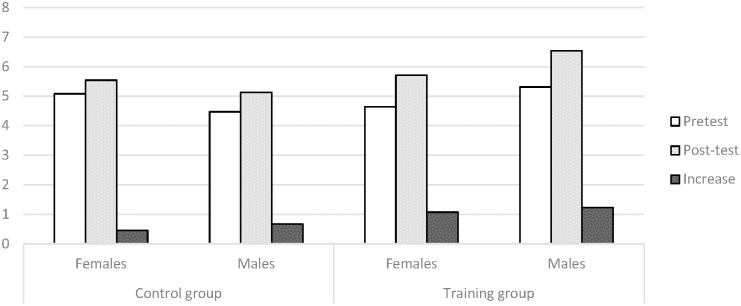
Mean scores for mental rotation in children in the control and experimental groups for the pretest phase, the post-test phase and the pre-post-test increases in 1st course of Preschool Education.

In **Figure 2**, the mean scores in MR for boys and girls are detailed and the pretest phase and the post-test phase, as well as the average increase for both are presented.

The interaction between group and sex found was due to the experimental design, as the groups were randomly created. Therefore, the results stated that no average sex differences would be found for this age group, either before or after training.

### Discussion

The main objective of this experiment was to assess whether the effect produced by a MR training can result in a higher performance of this spatial ability, measured through a different task to assess the participants’ level in such MR process in first course of Preschool Education. Our results suggest that it is not possible to assert the effectiveness of this training program in children aged between 3 and 4 years, being this result similar to that of other studies (Levine et al., 1999; Ehrlich et al., 2006; Frick et al., 2013a). However, it is noteworthy that, although both groups improved with respect to their pretest (likely due to the effect of learning and practice with the task), the increase in the training group was higher compared to the control group. Even though this increase was not significant, there was a tendency toward significance, which can mean that the results of the present study are congruent with a small effect of training on the experimental group. In this sense, the training may have been insufficient or not adjusted to this age group in relation to degrees of rotation that can be acquired at the Preschool stage. It could be that a more specific and intensive MR training could improve performance in a MR task through training in children aged between 3 and 4 years. Furthermore, the *Abbreviated Picture Rotation Test* could not have had a high enough index of discrimination, as a result of assessing only two angular degrees (45 and 180). Moreover, it is probable that some children had already acquired MR abilities while others had not, thus some children may have started to rotate in those angular discrepancies with greater ease thanks to this specific training.

With respect to sex differences, these data showed no difference between the group of boys and the group of girls, neither before nor after training. In order to investigate the possibility of finding sex differences, taking into account that training could modify these differences depending on the group, males and females were compared separately in each group, as previous studies have done (Sanz de Acedo Lizarraga and García Ganuza, 2003). Our results support those of other studies that have found no differences in the performance of MR tasks in children aged 3 and 4 years (Kaess, 1971; Platt and Cohen, 1981; Estes, 1998; Frick et al., 2013a).

Given the difficulty of establishing a clear training effect in first course of Preschool Education but with a tendency toward a higher improvement in those trained children, our Experiment 2 was focused on children 1 year older, corresponding to preschoolers in second course.

## Experiment 2

### Materials and Methods

#### Participants

Seventy one students participated in this experiment, although 10 children were excluded due to different reasons, such as: behavior problems or because the experimenter detected some difficulties in their performance across the tests. The final sample was composed by 61 children randomly distributed into training and control groups. The training group had 30 participants (14 boys and 16 girls; *M* = 3.90 and *SD* = 0.36), and the control group had 31 children (15 boys and 16 girls, *M* = 3.90 and *SD* = 0.35). The sample was recruited in same conditions as in Experiment 1, including the informed consent in accordance with the Declaration of Helsinki. This experiment was carried out in accordance with the recommendations of The Ethics committee of the University (UNED). The protocol was approved by the Ethics committee of the University (UNED).

#### Materials and Procedure

##### The Extended Picture Rotation Test

This test was adapted from the *Picture Rotation Test* (Quaiser-Pohl, 2003; Marke, 2008) to assess MR in 2nd year of Early Childhood Education. Although the original task was originally intended for use on 4–6 year-olds, 6 items of greater complexity were added to the original task, explained in Experiment 1, because this task was used in a previous experiment with older children (Fernández-Méndez et al., unpublished). In this way, the expanded version of the PRT had three practice tests and 22 assessment tests. The angles of rotation of the figures were 45°, 90°, 135°, 180°, 225°, 270°, and 315°.

The approximate duration of the task was 10–15 min, obtaining a point for each correct trial. The maximum score was 22 points. The test – retest reliability index was 0.63 in the trained group and 0.72 in the control group.

The Mental Rotation Training and the procedure carried out for 2nd course of Preschool Education were identical to those applied in Experiment 1.

### Results

The results of the MR scores in the control and training groups are shown in **Table 2**.

**Table 2:** Mean (standard deviation) and effect size (Cohen’s *d*) of mental rotation measures in the pretest and post-test and the pre-post-test differences (increases) for control group and training group in 2nd course of Preschool Education.

	Pretest	Post-test	Increase	*d*
Control group (*N* = 31)	11.42	15.17	3.26^∗^	
	(3.93)	(4.07)	(3.64)	
				*0.77
Training Group (*N* = 30)	11.20	17.07	5.87^∗^	
	(3.85)	(3.05)	(3.14)	

*^∗^*p* < 0.05, ^∗∗^*p* < 0.01.*

**FIGURE 3:**
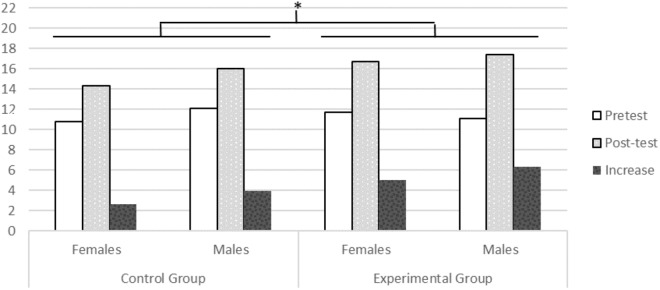
Mean scores for mental rotation in children in the control and experimental groups for the pretest phase, the post-test phase and the pre-post-test increases in 2nd course of Preschool Education. ^∗^Marks significant differences found between the training and control groups.

As in Experiment 1, a mixed-model repeated measures ANOVA was applied: 2 group (training vs. control) × 2 sex (males vs. females) × 2 time (pretest vs. post-test), with the first two factors as between-subjects and the last factor as within-subjects. The results revealed a significant main effect due to time, *F*(1,56) = 145.118, *p* = 0.000, $\eta_{p}^{2}$ = 0.72, and a significant interaction between time and group, *F*(1,56) = 8.337, *p* = 0.006, $\eta_{p}^{2}$ = 0.13. All effects involving the variable of sex were non-significant. In **Figure 3**, the mean scores in MR for boys and girls are detailed, the pretest phase and the post-test phase, as well as the average increase for both.

### Discussion

In contrast with Experiment 1, these results showed clear improvements in the performance of the training group compared to the control group in the MR task in preschoolers aged between 4 and 5 years old, after a training program. This study demonstrates how it is possible to improve MR ability in children of second year of Preschool Education, with a training designed to maximize this spatial skill with sufficient training trials. In spite of the results of previous studies (Levine et al., 1999; Ehrlich et al., 2006; Frick et al., 2013a), preschoolers under 5 years of age can be trained successfully in a MR task.

In relation to sex differences, there was no different between girls and boys either before or after training in any of the groups (control or training), similarly to Experiment 1. The data showed no evidence of male advantage in MR ability when assessed among preschoolers. Even though some studies have found evidence of superior performance of males in MR in preschoolers (Levine et al., 1999; Casey et al., 2008), others, such as the present study, maintain the emergence of a sex gap in posterior stages of development (Frick et al., 2009, 2013a).

## General Discussion

The main aim of this study was to test the improvement in MR ability after a MR training program in two courses of Preschool Education. Additionally, the differences between sex groups were analyzed. Spatial cognition research has shown the importance of spatial skills in a great number of activities of daily life. It is necessary to understand how they develop in such early stages of development as is the Preschool stage. Therefore, we have focused on the study of the development of MR in the 1st year (Experiment 1) and 2nd year (Experiment 2) of Preschool Stage.

Previous research studies have argued that children under 5 years of age (Frick et al., 2013a) or even 4 and a half years of age (Levine et al., 1999; Ehrlich et al., 2006) do not benefit from training in MR. However, the results of the present study refute these findings in children between 4 and 5 years old (Experiment 2). With respect to younger children, the results of Experiment 1 establish doubts regarding the possibility of effective training among children aged 3 and 4 years old, given the marginal training effect. The differential training effect found between Experiment 1 and 2 could be due to the test used to measure the MR training benefits in both courses. While the *Abbreviated Picture Rotation Test* had 8 items that tested an easy disparity (45 degrees of rotation) and the most difficult one (180degrees of rotation) to assess the improvement, the *Extended Picture Rotation Test* consisted in 22 items, in which 6 angular disparities (45, 90 135, 180, 225, 270, and 315 degrees of rotation) were tested. For this reason, although we can no assert the effectiveness of training in younger children, it is likely that more angular disparities, apart from 45° and 180° could discriminate among children with different levels of MR and be more sensitive to the training effect, making a distinction between trained and non-trained participants, given the results obtained with older children and the tendency toward significance in the younger ones. In spite of this explanation, the yielded results do not allow to assert that 3 year-old children could not successfully perform at other angular disparities beyond 45 and 180 degrees of rotation. Although the results of both experiments are not comparable, they suggest that the adequate age to train spatial skills, such as MR, could be from 4 years of age onward.

Furthermore, the differences found between our results and the studies that support the non-trainability of MR before 5 years of age may arise from the adaptation of the task to the level of performance of 3 and a half year olds, and to the larger number of sessions and trials with respect to Frick et al.’s (2013a) study. These authors only provided a single training session, with a total of 15 trials of MR plus 3 trials that comprised translation movements. Similarly, Ehrlich et al. (2006) and Marmor (1977) presented a training session with 32 (of which only 16 were rotations) and 12 trials (of which only 6 were rotations), respectively. The present study consisted of 3 sessions of increasing difficulty (with a total of 48 trials plus 14 practice trials), representing a substantial increase in training trials compared to previous research. The increased difficulty inter- and intra-session may have also meant that the participants learnt to perform rotations from simple angular disparities, favoring the learning process gradually with increasingly complex rotations (those with greater angular disparity). It is likely that the increased number of trials and sessions, with the gradual increase in difficulty, improved the performance of MR in children over 4 years of age, and even in 3-year-old children, which was reflected in a marginal increase in the training group with respect to the control group. The absence of effects in previous studies could be due to a maladjusted training, either because of the small number of trials or by not adapting the difficulty gradually, favoring the learning processes of MR. This study establishes, in contrast with previous studies, that preschool children can be trained following an adapted training with similar characteristics to those carried out in this work, thus, providing a solid foundation on which other studies can work on in the future. Probably, a longer intervention that the one performed in this study could increase performance more markedly in younger children, compared to that observed in this investigation. Moreover, it would be interesting to assess the maintenance of results over time. Considering the lack of studies that observe these effects over time in the Preschool stage, training may or may not have an effect after 3 or 6 months have elapsed.

Another point worth highlighting from this present study is the adaptation we performed of the original task by Quaiser-Pohl (2003), with the objective of evaluating the effect of MR training on 3 year-old children. In addition, the evaluation of the training program with a different task than that used during training supports the idea of knowledge transfer of spatial skills (Terlecki et al., 2008; Uttal et al., 2013a; Meneghetti et al., 2016a,b). In this regard, the earlier work of Marmor (1977), Ehrlich et al. (2006), and Krüger et al. (2014) did not assess transfer, as their training task was the same as their assessment test. These considerations favor research on the development and improvement of spatial skills within educational contexts, as it is a malleable and generalizable ability. Specifically, it can have potential repercussion in mathematical context, due to link between spatial ability and math (de Hevia and Spelke, 2010), where an early intervention is crucial to reduce the differences in mathematical performance (Jordan et al., 2009). In fact, Cheng and Mix (2014) showed an improvement in arithmetic problems after a MR training in children aged between 6 and 8, giving evidence of how promoting spatial ability can enhance math performance. Other studies have revealed that MR ability is a good predictor of math achievement in primary school children (Skagerlund and Träff, 2016), even Mix et al. (2016) proposed MR as the best predictor of mathematic performance in kindergarten. Since spatial skills are key to the success in STEM disciplines and fundamental for learning mathematics, spatial instruction has become a priority in education in early stages of development such as preschool, according to the National Council of Teachers of Mathematics (2006).

In relation to the average differences between sex groups, our results support those of other studies that have found no differences in the performance of MR tasks in children aged 3–5 years old (Kaess, 1971; Platt and Cohen, 1981; Frick et al., 2013a,b). It is noteworthy that MR training has improved this spatial ability in both sex groups of each age, which seems to consolidate the idea that the appearance of average sex differences in MR between groups occurs after the Early Childhood Education stage (Johnson and Meade, 1987; Titze et al., 2010) and is not innately found at the start of development. The present study emphasizes the possibility of maintaining equality between sex groups, as sex differences (favoring males) are well established in adulthood (Linn and Petersen, 1985; Voyer et al., 1995), as well as the need to know the critical stage at which these differences begin to be noticeable. For this reason, further research of both the origin and the development of sex differences in terms of spatial ability in 3rd course of Preschool Education are required. It could be hypothesized that the superior performance exhibited by men in later stages than those studied in this research obey more to an educational and social influence linked to sex, than to a difference according to sex *per se*. It is likely that between 3 and 5 years of age, children still have not been differentially exposed to spatial experiences that could be influencing the observed spatial performance at later stages (Sander et al., 2010). Actually, a recent work on MR training with teenagers found no sex differences before or after training for spatial ability (Rodán et al., 2016).

In sum, taking both statements together (both the existence of MR malleability, and the absence of differences between girls and boys of this age), it is important to note the possibilities of implementing actions within the education system to enable the equal development of spatial skills in both sexes. This equal and non-discriminative development could result in a greater representation of females within STEM disciplines in the future, given their relationship with spatial skills and the lower presence of women in relation to men nowadays.

It should be highlighted that this study is, to our knowledge and according to the review of the literature performed, the first that has shown a marginal MR training effect on children in 1st year of Preschool Education and a clear effect on 2nd year of Preschool children. It is also remarkable that this training effect was evaluated through a different task than that used in training, thereby proving a transfer of the obtained learning to untrained tasks.

## Limitations and Future Research

This study suffers some limitations that must be highlighted in order to understand the reaching as well as the restrictions derived from the conclusions. Considering both, we can establish future lines of research to promote the MR ability in preschoolers in an accurate way.

The principal limitation is referred to the *Abbreviated Picture Rotation Test* used to assess the improvement due to training in 3 and 4 year-old children. Although we followed the recommendation of the test’s author, it was not enough to ensure its adequacy. The test comprised 8 items, where only two angular disparities were tested, whereas 6 different rotations had been trained. Also, the items had two response options with the risk of performing at chance level. Together, the test may have lost discrimination capacity by not including more angular disparities or more response options.

Also, the use of an active control group would have been recommendable in order to disentangle between specific and general (improvements due to motivation and/or engagement) training effects. However, the fact of having a control group is a methodological improvement with respect to previous training research in which spatial ability was trained without a control group (Verner, 2004; Rafi et al., 2008). The lack of a control group is an important limitation in training studies due to difficulty of distinguishing between test-retest effect and training effect (Uttal et al., 2013a). This limitation is surpassed in this present study by including a comparison control group.

Furthermore, the same version of the PRT was administered in the pre and post-test, and, as a consequence, the children could have experienced an effect of test–retest practice in the control and training groups. For this reason, it is important to have at least one control group in order to show training effect separately from learning effect for test-retest practice. Finally, although a larger sample would have been desirable for greater representation of the results, it is not usual to find studies of this type with much larger samples than that of the present work. Specifically, this work was performed over a period of 2 years. The assessment had to be child by child and each session was brief in order to avoid fatigue and to maintain the child’s attention, given the children’s age.

Another limitation of the present study was not including a general cognitive control measure such as working memory or reasoning ability to match control and training groups. However, it is important to note that both groups were randomly created and no differences were detected between them in their MR ability before the training phase.

Future research studies that intend to improve MR ability during the first course of preschool stage must consider using another test to assess the benefit due to training, examining the limits observed in the test used here. As it can be observed, the extended PRT was used for the second course of Preschool with a good performance. For this reason, the original PRT with 16 experimental items and three response options could be used to assess MR ability in children between 3 and 4 years old. Nevertheless, we would recommend performing a pilot study before using it in future works with an extended sample.

## Author Contributions

All authors: conceived and designed the experiment, interpretation of the data, and contribution to the redaction. LF-M: performed the experiment, analyzed the data, and drafted the paper. MC and ME: provided critical revision. All authors: approved the final version of the paper for submission.

## Conflict of Interest Statement

The authors declare that the research was conducted in the absence of any commercial or financial relationships that could be construed as a potential conflict of interest.

## Footnotes

1 Throughout the article, we have used the term “sex differences” to refer to both, biological and psychological aspects of the group differences between males and females in cognitive abilities, as Halpern (2012) prefers, instead of using “gender differences.” In the present study, we have categorized boys and girls based on biology; children were not asked about their adherence to gender roles.
